# Stand if you can- A parallel, superiority cluster randomized controlled trial to improve gait speed for long term care residents

**DOI:** 10.1016/j.jarlif.2025.100015

**Published:** 2025-05-09

**Authors:** Kendra Cooling, Danielle R. Bouchard, Molly Gallibois, Jeffrey Hebert, Martin Sénéchal, Pamela Jarrett, Chris McGibbon, Emily Richard, Grant Handrigan

**Affiliations:** a Faculty of Kinesiology, University of New Brunswick Fredericton New Brunswick Canada; b Cardiometabolic Exercise & Lifestyle Laboratory, Fredericton, New Brunswick, Canada; c Horizon Health Network, Saint-John New Brunswick Canada; d Faculty of Nursing, University of New Brunswick, Moncton New Brunswick Canada; e École de kinésiologie et de loisir, Université de Moncton, Moncton, New Brunswick Canada

**Keywords:** Gait speed, Standing, Older adults, RCT, LTC

## Abstract

**Objective:**

To examine the effects of a standing intervention on gait speed for older adults living in long term care (LTC) residences.

**Design:**

A parallel superiority cluster randomized controlled trial.

**Setting and participants:**

LTC residences. A total of 95 LTC residents (n = 47 control; n = 48 intervention) participated in the study.

**Methods:**

LTC residences and therefore the residents from the homes were randomized to either the intervention group (standing up to 100 minutes/week) for 22 weeks or the control group (socializing with staff with no encouragement to stand for up to 100 minutes/week) for 22 weeks. The primary outcome is gait speed measured by the 10-meter walking speed test.

**Results:**

A total of 95 participants (n= 47 in the control group and n=48 in the intervention group) age 86 ± 8 years completed the trial, averaging 41.9 ± 30.3 min of standing per week in the intervention group and 48.4 ± 22.8 min of time matched activity in the control group. There was no significant difference between groups in changes in gait speed (β=-0.034, 95 % C.I. (-0.097 0.028)).

**Conclusions and implications:**

This 22-week standing intervention did not improve gait speed in older adults living in LTC residences.

Trial registration: clinicaltrials.gov - NCT03796039

## 1 Introduction

Currently, 30 % of Canadians 85 years and older are living in long-term care (LTC), which includes nursing homes and residential care [1]. Many of these individuals have reduced gait speed [2], which is a key component of physical function. Gait speed is considered of such importance that it has been referred to as the *sixth vital sign* [3]*.* A gait speed lower than 1.0 m/s is associated with a loss of independence and an increased risk of hospitalization [4]. Studies have shown, for each 0.10 m/s reduction in baseline gait speed there is a 10 % decrease in functional status [3,5,6]. The rate at which gait speed decreases is also linked to mortality and decreased physical function, with faster declining changes in gait speed associated with increased mortality [6]. Also, there is a strong association between slow walking speed and cardiovascular mortality in older adults [7]. Therefore, gait speed appears to be a potential indicator of physical function, risk of hospitalizations and a predictor of mortality. As a result, identifying strategies for maintaining, or potentially improving the gait speed of older adults in LTC may lead to improved physical function, greater independence and lower mortality rates [5].

Traditional exercise such as walking or muscle strengthening activities have shown to improve gait speed for residents in LTC settings [[8], [9], [10], [11]]. However, these interventions often exclude people with high frailty scores, or those with significant cognitive impairments. Recently, it has been demonstrated that light intensity activities such as standing could potentially be linked to health and functional ability of older adults [12]. There is potential for positive physical changes using a multilevel intervention aimed at reducing sitting time [13]. Further, a large scale epidemiological study suggests that replacing three hours of sitting time with standing led to a potential decrease of 12 to 18 % in all-cause mortality risk [14]. Because of the potential benefits of performing light intensity physical activity there is interest to evaluate the effectiveness of interventions that reduce sedentary time and increase light intensity physical activity in older adults. Standing is a light intensity physical activity that is appealing in its simplicity. It is possible that there are potential benefits, as measured by gait speed, for older adults who practice standing as part of a physical intervention.

Therefore, the *Stand if You Can* randomized controlled trial (RCT) aimed to compare the effects of a standing intervention targeting the average gait speed in the intervention group compared to a control group that matched for social exposure time. It was hypothesized that adding 100 min of standing per 5-day week for 22 weeks would significantly improve gait speed of participants in the intervention group compared with participants in the control group receiving the same time social exposure without any encouragement to stand.

## 2 Methods

### 2.1 Trial design

This study was a multi-site parallel cluster randomized controlled trial (NCT03796039, clinicaltrials.gov), designed to evaluate the change in gait speed of older adults living in LTC residences. The intervention consisted of standing for up to 100 min per week for 22 weeks and the control group received matched social exposure time without any encouragement to stand.

### 2.2 Participants

**Fig. 1:**
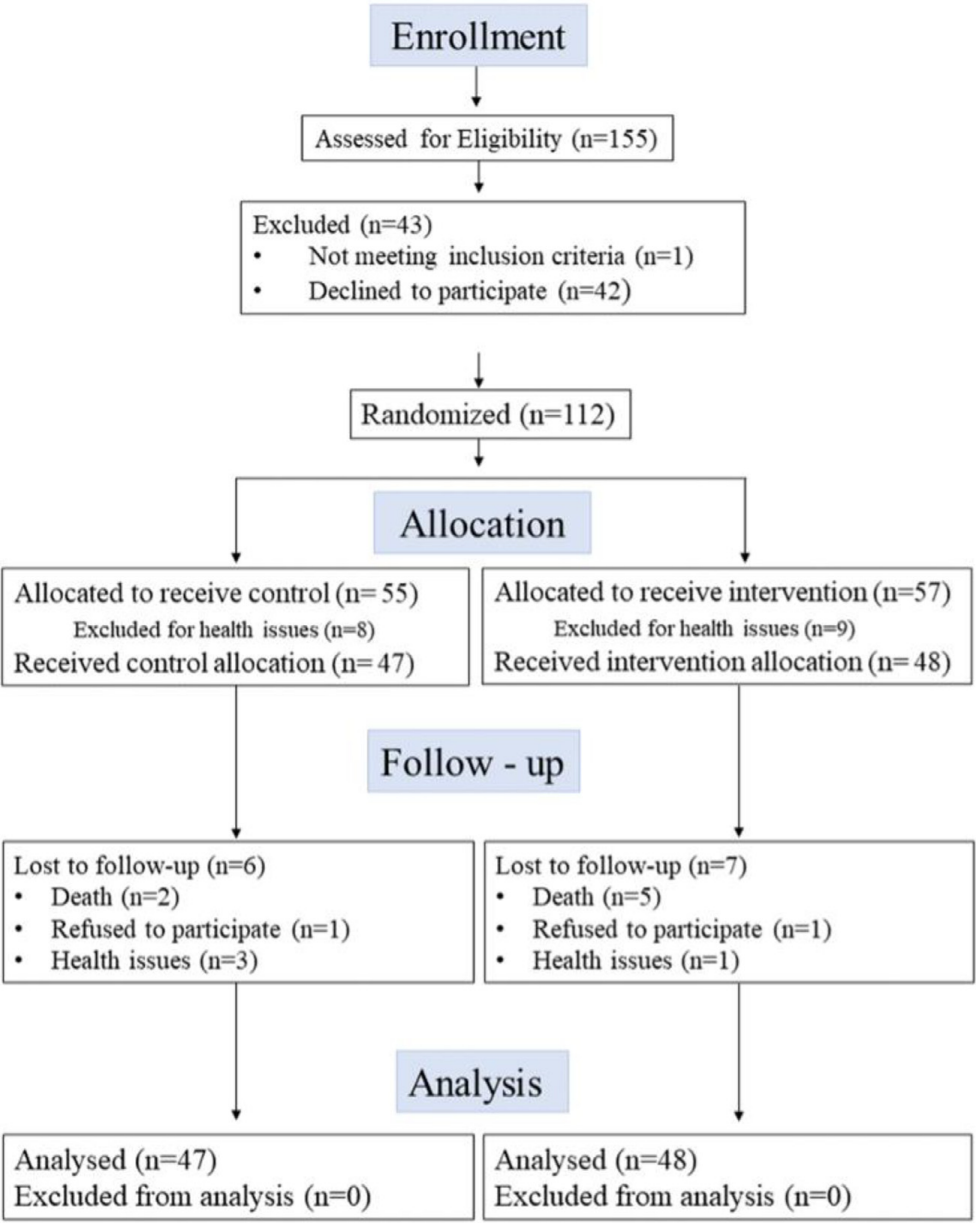
Participant flow chart.

A total of four long-term care settings with a minimum occupancy of 100 residents were recruited to participate. Two homes were randomly allocated to the active intervention group and the other two homes allocated to the control intervention group. Active intervention and control intervention sites were concealed to research staff and participants until after the baseline evaluations were performed. To participate in the study, residents of the LTC homes had to meet the following inclusion criteria: to be a resident of one of the residences; able to provide consent or have a substitute decision maker agree on their behalf to participate; able to walk for ten meters, with or without a walking aid. Individuals were excluded from the study if they were unable to meet the inclusion criteria, or they were deemed too high risk for falling by the residence staff. Potential participants were identified by the LTC staff and family members in collaboration with the research staff and the staff at the home approached participants for their potential interest. After a potential participant was identified, research assistants provided the participant and/or the substituted decision maker with the details of the study and answered any questions before obtaining consent. The participant flow chart in Fig. 1 identifies the recruitment process and participant allocation [15].

Demographic data such as age, weight and height, and length of stay in the residence were provided by the residences at the start of the trial. Frailty status was determined by nursing staff in the residence using the Clinical Frailty Scale [16]. Research staff performed the Mini Mental State Examination [17].

### 2.3 Intervention

This study included two intervention arms, the active intervention group, and the control intervention group. In the active intervention group, participants were encouraged and assisted, when necessary, to stand for up to an additional 100 min per week, outside of their usual activities, for 22 consecutive weeks. The active intervention was implemented in two 10-minute periods per day (morning and afternoon), from Monday to Friday, for 22 weeks and was based on results from a pilot study [18]. Participants were always assisted by a research assistant and stood either individually, or in small groups. Each participant was permitted to take breaks as needed during the standing session. Up to five attempts to stand and maintain a standing position were permitted until the research assistants no longer provided encouragement to stand. Participants were permitted and encouraged to use their usual standing assists such as walkers or canes for stabilization during standing.

The control intervention group received time-matched visits from research assistants, specifically one daily 20-minute visit five days per week for a total of 100 weekly min for 22 consecutive weeks. During these visits the research assistants did not encourage or discourage participants to stand and spent time with the participants in the settings and activities that were ongoing upon visitation.

For both intervention arms, during the time with the participants, the research assistants discussed current issues, the weather or performed social activities such as board games, painting and listening to music. All contact time with participants in both groups was recorded using stopwatches and pencil and paper logs for the entire duration of the intervention.

### 2.4 Primary outcome

All outcomes were measured at the LTC residence pre and post intervention, unless otherwise noted. Gait speed was the primary outcome using a validated 10-meter walking test [3,[19], [20], [21]]. Two meters at the beginning and end of the 10-meter walking distance were removed to account for acceleration and deceleration phases. Walking speed was therefore measured with a stopwatch over a six-meter walking distance. Participants were encouraged to use their usual walking assists, as needed. Each participant performed the test twice, the average of both tests was used to determine average gait speed for all participants. Participants were instructed to walk at their normal gait speed for the entire 10-meter distance.

### 2.5 Sample size estimation

The primary outcome in this RCT was gait speed. With a cluster size of two, and assuming a small-to-moderate clustering effect (intraclass correlation coefficient = 0.075), a within-group standard deviation of 0.10 m/s [18,22,23], an alpha = 0.05, and 80 % power, it was determined that this study would require at least 36 participants per group. To account for an expected loss to follow-up of 35 %, 48 participants per group were recruited. Recruitment was completed when 48 eligible participants per group were evaluated and deemed suitable for the study.

## 3 Randomization

### 3.1 Sequence generation

Sequence generation was performed using a randomization generating website, sealedenvelope.com. A block size of two and two treatment groups, active intervention and control intervention, were used to generate the allocation sequence. Four sites, separated as two clusters (active or control interventions), were allocated to the randomization. Allocation was concealed at the cluster level until all groups were evaluated for baseline measures. A research colleague not active in the trial implementation was assigned the randomization process, and kept allocation concealed until all baseline data was collected.

### 3.2 Blinding

Participants, caregivers, and study staff were blinded prior to group allocation; however, all study staff and participants were unblinded for post testing.

### 3.3 Statistical Methods

Statistical analyses were performed using R (4.1.0). Descriptive statistics were used to measure the characteristics of the participants, and results are presented as mean ± SD. As this is a randomized controlled trial, an intention to treat (ITT) approach was used [24]. Treatment effects were estimated using a random-intercept linear mixed model with six-meter gait speed as the outcome variable. Consistent with an intention to treat approach, the model applied maximum likelihood estimation to handle missing data, and all participants randomized to the intervention groups were included in the analyses of outcomes. A linear mixed-effects model was used to assess the effect of the intervention on gait speed, adjusting for baseline gait speed and accounting for individual-level repeated measures. The model included fixed effects for group (active intervention vs. control), time (pre- and post-intervention), their interaction, baseline gait speed, and cluster. A random intercept was included for participant ID to account for the within-subject correlation due to repeated measurements. The model is specified as:

Avg_Gait ∼ Group * phase + baseline_gait + cluster + (1 | ID).

In this model, cluster was treated as a fixed effect to control for any systematic differences between the four clusters, while still estimating the within-subject variability. Adjusting for baseline gait speed improves precision in estimating the treatment effect by accounting for initial differences in gait performance. The hypothesis of interest was the group by time interaction. The linear mixed model analysis was performed using the ‘lme4’ package (v1.1-28) [25].

## 4 Results

A total of 155 residents were assessed for eligibility (Fig. 1) and 95 were randomized within the four LTC residences: 47 in the control group (29 women, 61.7 %) in two residences and 48 in the intervention group (39 women, 81.2 %) in two other residences. In total, six participants did not complete their participation in the control group and seven participants in the intervention group. These participants withdrew for a variety of reasons, including health, mortality, and lack of interest (Fig. 1). Baseline characteristics of randomized participants are presented in Table 1. For the control intervention group, the average age of participants was 85.3 ± 7.7 years, 31/47 (65.9 %) were female and scored an average MMSE of 18.2 ± 8.4. For the active intervention group, the average age was 87.6 ± 8.3 years, 36/48 (75 %) were female and scored an average MMSE of 16.8 ± 8.1.

**Table 1:** Baseline characteristics of participants.

Characteristics	Control Group (n=47)	Intervention Group (n=48)
**Age (years)**	85.3 ± 7.7	87.6 ± 8.26
**Sex (female, n)**	31	36
**BMI (kg/m^2^)**	25.5 ± 8.9	26.8 ± 6.6
**Clinical Frailty Scale (1-9)**	6.15 ± 0.8	6.0 ± 1.5
**Fit (1-3)** **Moderate Frailty (4-6)** **Severe Frailty (7-9)** **Transfer status**^a^ **Independent transfer** **Assist Transfer** **Dependent Transfer** **MMSE Score (0-30)**^b^ **Mild (24-30)** **Moderate (18-23)** **Severe (0-17)**	0 30 17 25 13 9 18.2 ± 8.4 15 14 18	6 15 27 7 30 11 16.8 ± 8.1 13 8 24
**Length of time living in LTC residence (months)**	45.8 ± 41.7	56.4 ± 62.1

a values shown are number of participants per transfer classification status.

b N = 45 for the MMSE in the intervention group due to missing data.

Participants were encouraged to stand for up to 100 min per week for 22 weeks. On average, participants stood 42.4 ± 30.8 min per week.

**Fig. 2:**
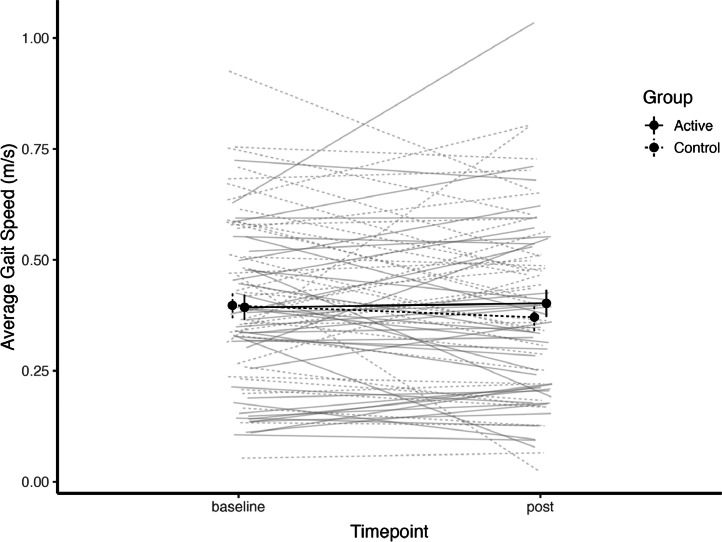
Average gait speed (± 95 % CI) for the participants in both groups at both timepoints. The control intervention group are represented by the solid line and the active intervention are the dashed line. Individual-level change lines are presented for all participants.

Fig. 2 displays the pre and post gait speed measures for both groups. The average gait speed went from 0.35 m/s (95 % C.I. (0.30, 0.41)) to 0.36 m/s (95 % C.I. (0.30, 0.42)) in the active intervention group and from 0.44 m/s (95 % C.I. (0.39, 0.50)) to 0.42 m/s (95 % C.I. (0.36, 0.47)) in the control intervention group. The results of the random-intercept linear mixed model analysis revealed when adjusted for baseline gait speed, that the group*time interaction (GroupStand:phasepost) was not statistically significant (Estimate = 0.0359, t = 1.227, p > 0.05). This suggests that the active intervention group showed a slight increase in gait speed relative to the control group from pre- to post-intervention, but this effect did not reach statistical significance.

The interaction effect (GroupStand:phasepost) was positive, aligning with the hypothesis that the intervention group would show an improvement in gait speed.

There were no serious adverse events to report in this trial. However, there were three non-injurious falls in three participants during the delivery of the standing interventions for the active intervention group.

## 5 Discussion

The primary objective of this study was to measure changes in gait speed of older adults who participated in a light intensity physical activity intervention that encouraged participants to stand up to 100 min per week for 22 weeks, compared to a control (exposure time matched) intervention group who did not receive encouragement to stand. The main analysis revealed that there were no statistical differences found between the two groups with respect to improvement in gait speed. Therefore, the standing intervention did not have a statistically significant impact on gait speed performance. There are several possible reasons that may explain this including intervention exposure and participation and a lack of consensus on what is a minimally clinically important difference (MCID) for this population. As well, this population of older adults have a high prevalence of cognitive impairment and other physical illnesses that likely also impact their ability to participate fully and benefit from this intervention.

On average, participants did not attain 100 min of weekly standing. In fact, participants performed approximately 42 min of standing time per week. This is despite participants being encouraged to stand twice daily five times per week for up to 10 min per session for the entire 22-week intervention period. Therefore, a lack of encouragement and opportunity does not appear to be a likely cause for this inability to attain the standing time goal of 100 min of weekly standing time. The participants in the active intervention group all had the opportunity to stand up to 100 min per week, but on average only stood for less than half of this time. This suggests that the dose of the light intensity physical activity intervention was appropriate for the participants. It was neither too high, nor too low.

In the literature, there is a lack of consensus on a MCID for gait speed in older adults who are frail. Also, it has been suggested that in clinical trials sample size and variability can impact the ability to determine statistical significance [26] and that interventional effects can be evaluated by comparison with meaningful differences [27]. A commonly cited MCID for gait speed for community-dwelling adults 65 and older is 0.1 m/s [23,28,29]^,^ although smaller changes are considered meaningful, such as 0.03 to 0.06 m/s and substantial change is considered 0.1 m/s [27,30]. Further, a 2013 Cochrane systematic review found a fixed pooled effects of exercise on LTC residents’ gait speed of 0.03 m/s (95 % CI=0.00, 0.07) [31]. Also of note, it is normal to experience decreases in gait speed in older adults between the ages of 70–79 of approximately -0.02 m/s for men and -0.03 m/s for women annually, suggesting a typical rate of change is a decrease in gait speed with aging [6]. This normal decrease likely continues with aging. In this study, the actual differences between active and control intervention groups are similar to small meaningful changes reported in other studies. Coupled with evidence that older adults benefit significantly from engaging in physical activity even when well below the recommended level of activity [32] and reducing sedentary behaviors [14], the gait speed results are conceivably clinically important. Overall, there is limited understanding on the clinical significance for improving gait speed for older adults living in LTC residences. Therefore, in the absence of an established MCID, it is difficult to provide any clinical interpretation of gait speed outcomes in this study.

This study includes several features that set it apart from typical exercise research in older adults, particularly in terms of its inclusivity. Specifically, participants were not excluded from this study due to cognitive impairment. Almost all participants had an MMSE score of 24 or below and an average of between 16-18 in both groups. Scores of 16-18 indicate at least a moderate degree of cognitive impairment [33]. This created a challenge for the evaluations and interventions as participants periodically had difficulty to follow instructions. Also, an intention-to-treat approach was used meaning that all participants were included in the analyses despite the large variability in participation that occurred. Therefore, the outcomes in this study may have been influenced by cognitive status and participation.

## 6 Limitations

In addition to the challenge that the presence of cognitive impairment in the study population created, this study has other limitations, including that research assistants were not blinded to participant group allocation at post intervention evaluation. Also, this was a single blind intervention, and the participants were aware of which group allocation they received, as is typical with exercise interventions [34]. Further, caution is warranted in interpreting the results of this study due to the small sample size and small number of clusters. Finally, this study may be subject to consent bias, as individuals who chose to participate could differ in key ways from those who declined, potentially limiting generalizability.

## 7 Conclusions and implications

In summary, the implementation of a standing program did not result in a statistically significant improvement in the gait speed for residents of LTC homes. However, there is evidence for small MCID in gait speed in older adults, but the clinical significance of small gait speed changes in this population is not well established. Further exploration may be required to understand whether intervening at earlier stages of care could impact physical function determinants such as gait speed, as well as the trajectory of gait speed and mortality, for residents of LTC homes.

## CRediT authorship contribution statement

**Kendra Cooling:** Writing – review & editing, Writing – original draft, Project administration, Investigation, Formal analysis, Data curation. **Danielle R. Bouchard:** Writing – review & editing, Writing – original draft, Supervision, Project administration, Methodology, Investigation, Funding acquisition, Data curation, Conceptualization. **Molly Gallibois:** Writing – review & editing, Project administration, Data curation. **Jeffrey Hebert:** Writing – review & editing, Methodology, Funding acquisition. **Martin Sénéchal:** Writing – review & editing, Methodology, Formal analysis. **Pamela Jarrett:** Writing – review & editing, Methodology, Funding acquisition, Formal analysis. **Chris McGibbon:** Writing – review & editing, Methodology, Funding acquisition, Formal analysis. **Emily Richard:** Writing – review & editing, Methodology, Funding acquisition, Formal analysis. **Grant Handrigan:** Writing – review & editing, Writing – original draft, Project administration, Methodology, Investigation, Funding acquisition, Formal analysis, Data curation, Conceptualization.

## Declaration of competing interest

The authors declare that they have no known competing financial interests or personal relationships that could have appeared to influence the work reported in this paper.

The authors declare the following financial interests/personal relationships which may be considered as potential competing interests:

Grant Handrigan reports financial support was provided by Canadian Frailty Network. Danielle Bouchard reports financial support was provided by Canadian Frailty Network. If there are other authors, they declare that they have no known competing financial interests or personal relationships that could have appeared to influence the work reported in this paper.
